# Downregulation of miR-451 in cholangiocarcinoma help the diagnsosi and promotes tumor progression

**DOI:** 10.1186/s12860-022-00445-2

**Published:** 2022-11-09

**Authors:** Dengfang Guo, Qingling Wang, Jiancheng Huang, Zhanglin Hu, Chun Chen, Chun Zhang, Feng Lin

**Affiliations:** 1 Department of General Surgery, Mindong Hospital Affiliated to Fujian Medical University, 89 Heshan Road, 355000 Ningde, Fujian China; 2 Department of Medical Laboratory, Mindong Hospital Affiliated to Fujian Medical University, 355000 Ningde, Fujian China

**Keywords:** miR-451, Cholangiocarcinoma, Early detection, Prognosis prediction, Biological function

## Abstract

**Background:**

Cholangiocarcinoma is a kind of invasive malignant tumor followed by hepatocellular carcinoma. miR-451 was suggested to function as regulator in various human tumors, but its role in mediating tumor progression and predicting the prognosis of cholangiocarcinoma remains unknown. The clinical significance and biological function of miR-451 in cholangiocarcinoma were assessed in this study.

**Results:**

The tissue and serum expression of miR-451 was decreased in cholangiocarcinoma compared with corresponding normal samples. The downregulation of miR-451 was associated with the progressive TNM stage and positive lymph node metastasis of patients. miR-451 was identified to be an indicator of the diagnosis and prognosis of cholangiocarcinoma distinguishing cholangiocarcinoma patients from healthy volunteers and predicting the poor outcome of patients. miR-451 also served as a tumor suppressor negatively regulating the cellular processes of cholangiocarcinoma.

**Conclusions:**

miR-451 played a vital role in the early detection and risk prediction of cholangiocarcinoma. miR-451 also suppressed the progression of cholangiocarcinoma, which provides a potential therapeutical target for cholangiocarcinoma treatment.

**Supplementary Information:**

The online version contains supplementary material available at 10.1186/s12860-022-00445-2.

## Background

Cholangiocarcinoma is a malignant tumor in the hepatic second to hepatocellular carcinoma. Cholangiocarcinoma is a group of epithelial cancers mentioning the intrahepatic, perihilar, and distal biliary tree [1]. Owing to the invasive characteristics of cholangiocarcinoma, the disease development is uncontrolled, and there was a lack of obvious clinical characteristics and risk factors, which makes patients always diagnosed at an advanced stage [2]. Although the diagnosis and therapy technology have been developed, the incidence and mortality of cholangiocarcinoma are still increasing [3]. Identifying effective biomarkers to diagnose cholangiocarcinoma at an early stage and predict disease development could ameliorate patients’ clinical outcomes and improve the cure rate of cholangiocarcinoma.

microRNAs (miRNAs) have been demonstrated to serve as indicators in the diagnosis, prognosis, and progression of human cancers [4]. Binding with the 3’UTR of relevant mRNAs is the major characteristic of miRNA, by which miRNAs mediate the cycle progression, apoptosis, and growth of cancer cells, and therefore participate in the occurrence and development of tumors [5]. The dysregulation of different miRNAs always implies their functional role in human diseases. For example, increased miR-25 was correlated with malignant development and poor survival of cholangiocarcinoma patients [6]. miR-186 was disclosed to be downregulated and suppress the proliferation, migration, and invasion of cholangiocarcinoma cells [7]. In the previous identification of differently expressed miRNAs which were considered candidate biomarkers of cholangiocarcinoma progression, miR-451 was found to be downregulated [8]. It has been reported that miR-451 not only regulated the biological function of tumor cells, but also regulated the physiological and pathological processes of humans, and it was also considered a novel therapeutic target of human cancers [9, 10]. miR-451 also shows significant diagnostic value in ischemic stroke and papillary thyroid carcinoma [11, 12]. In colorectal cancer, miR-451 inhibited cell growth and metastasis via targeting MIF [13]. While the specific function of miR-451 remains unclear.

This study aimed to validate the expression of miRR-451 in cholangiocarcinoma and disclose its potential in clinical diagnosis and risk prediction of cholangiocarcinoma.

## Results

### miR-451 was downregulated in cholangiocarcinoma and was associated with patients’ clinical features

**Fig. 1:**
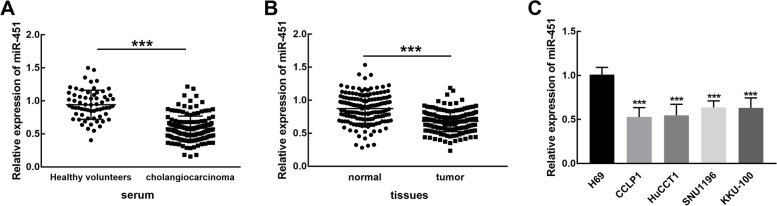
The expression levels of miR-451 in cholangiocarcinoma. **A** The expression of miR-451 was significantly lower in the serum of cholangiocarcinoma than that of healthy volunteers. **B** miR-451 was significantly downregulated in tumor tissues of cholangiocarcinoma patients in comparison with matched normal tissues. **C** miR-451 was downregulated in cholangiocarcinoma cell lines than normal cells. ***P < 0.001

The expression of miR-451 was significantly lower in the serum of cholangiocarcinoma than that in the serum of healthy volunteers (*P* < 0.001, Fig. 1A). In the collected tissues, miR-451 was significantly downregulated in tumor tissues in comparison with the matched normal tissues (*P* < 0.001, Fig. 1B). Consistently in cholangiocarcinoma cell lines, the downregulation of miR-451 was also observed and showed a dramatic difference with normal cells (*P* < 0.001, Fig. 1C).

Patients were partitioned into a high miR-451 group and a low miR-451 group based on the average expression level of miR-451 in serum and tissues of cholangiocarcinoma. The relatively low expression of miR-451 in tissues showed a significant association with the TNM stage (*P* = 0.014) and lymph node metastasis status (*P* = 0.015) of patients (Table 1). Consistently, a close association was also found between the serum miR-451 expression and the TNM stage (*P* = 0.022) and lymph node metastasis status (*P* = 0.042) of patients (Table 1).

**Table 1:** Association between miR-451 expression and clinical features of patients

	Total (n = 159)	miR-451 in tissues		*P* value	Total (*n* = 159)	miR-451 in serum		*P* value
		Low (*n* = 83)	High (*n* = 76)			Low (*n* = 82)	High (*n* = 77)	
Age				0.566				0.593
< 60	82	41	41		84	45	39	
≥ 60	77	42	35		75	37	38	
Gender				0.883				0.347
Male	89	46	43		91	44	47	
Female	70	37	33		68	38	30	
TNM stage				0.014				0.022
I-II	95	42	53		97	43	54	
III-IV	64	41	23		62	39	23	
Differentiation				0.196				0.262
Well-moderate	92	44	48		94	45	49	
Poor	67	39	28		65	37	28	
LNM				0.015				0.042
Negative	104	47	57		103	47	56	
Positive	55	36	19		56	35	21	
location				0.297				0.373
Intrahepatic	81	39	42		83	40	43	
Extrahepatic	78	44	34		76	42	34	

*LNM* Lymph node metastasis

### miR-451 was identified as a biomarker for the diagnosis and prognosis of cholangiocarcinoma

**Fig. 2:**
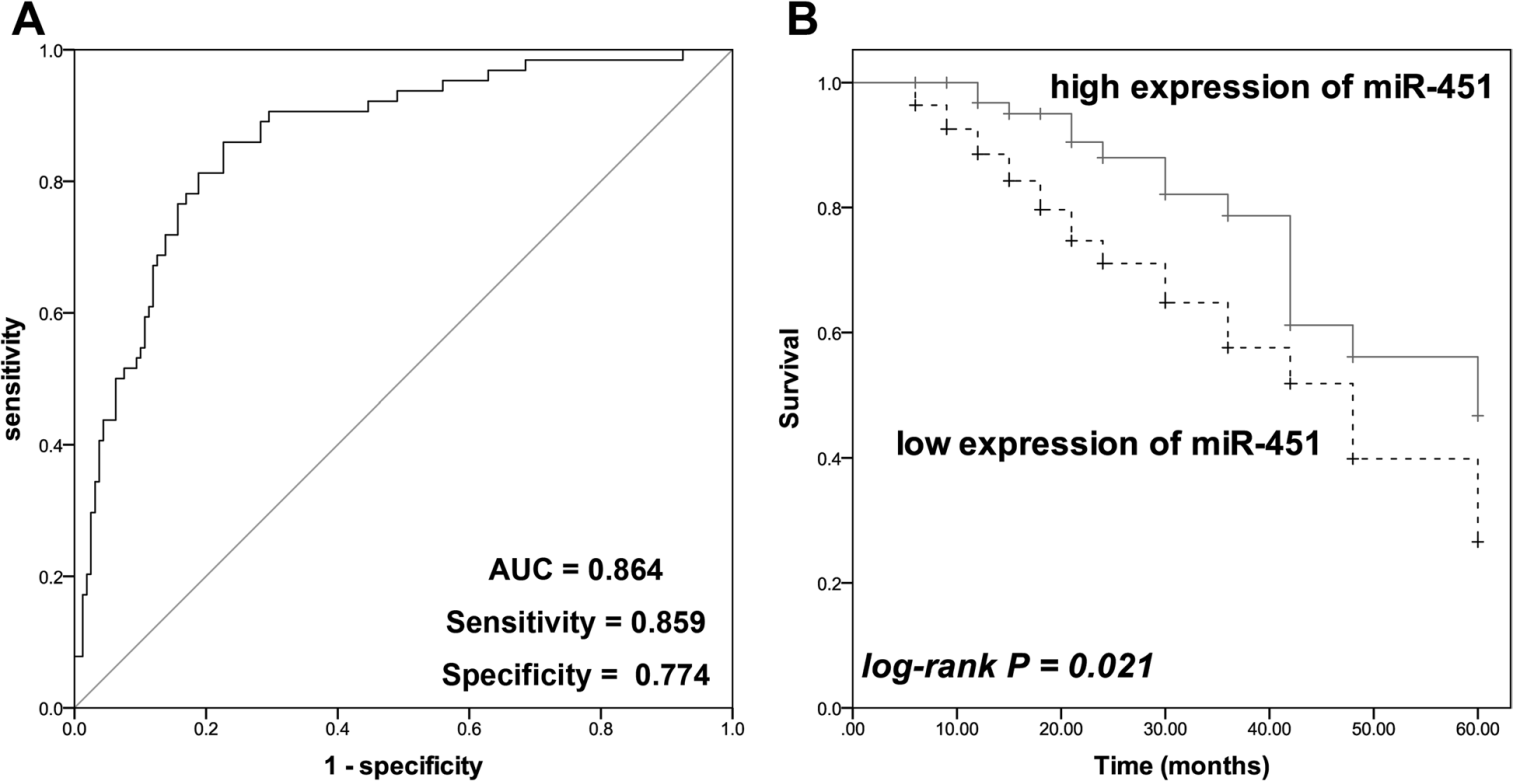
The clinical significance assessment of miR-451. **A** miR-451 could differentiate cholangiocarcinoma patients from healthy volunteers with the AUC of the ROC curve of 0.864. **B** The relatively low expression of miR-451 was associated with the poor survival of cholangiocarcinoma patients. log-rank *P* = 0.021

miR-451 could distinguish cholangiocarcinoma patients from healthy volunteers with the AUC value of 0.864 of the ROC curve (sensitivity = 0.859, specificity = 0.774, Fig. 2A). Additionally, in cholangiocarcinoma patients, the downregulation of mir-451 was associated with the worse survival of patients (log-rank *P* = 0.021, Fig. 2B). Moreover, Cox regression analysis further demonstrated the prognostic value of miR-451. miR-451 and the TNM stage served as independent prognostic indicators of patients with HR values of 2.651 and 2.277, respectively (Table 2).

**Table 2:** Association between clinical features and survival of patients by Cox regression analysis

	HR value	95% CI	*P* value
miR-451	2.651	1.397–5.029	0.003
Age	1.063	0.575–1.965	0.844
Gender	1.262	0.708–2.252	0.430
TNM stage	2.277	1.147–4.518	0.019
Differentiation	1.354	0.744–2.466	0.321
LNM	1.717	0.840–3.509	0.138
location	1.342	0.740–2.431	0.333

*miR-451* miR-451 expression in cholangiocarcinoma tissues; *LNM* Lymph node metastasis

### miR-451 suppressed the biological processes of cholangiocarcinoma cells

**Fig. 3:**
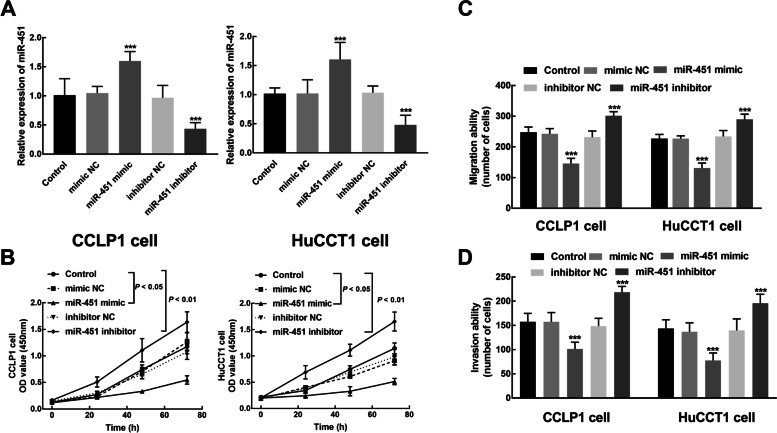
Transfection efficiency evaluation in CCLP1 and HuCCT1 cells (**A**). miR-451 was dramatically overexpressed by the transfection of miR-451 mimic and silenced by the transfection of miR-451 inhibitor. The biological function of miR-451 evaluation in CCLP1 and HuCCT1 cells. **B** The proliferation of CCLP1 and HuCCT1 cells was significantly promoted by miR-451 knockdown and suppressed by the overexpression of miR-451. **C** The upregulation of miR-451 dramatically inhibited the migration of CCLP1 and HuCCT1 cells, while its downregulation remarkably enhanced the cell migration of cholangiocarcinoma. **D** The upregulation of miR-451 dramatically inhibited the invasion of CCLP1 and HuCCT1 cells, while its downregulation remarkably enhanced the cell invasion of cholangiocarcinoma. ***P < 0.001

Due to the relatively high sensitivity of CCLP1 and HuCCT1 cells to the downregulation of miR-451, these two cells were selected for the following *in vitro* cell experiments. miR-451 was overexpressed by the transfection of miR-451 mimic and silenced by the transfection of miR-451 inhibitor in CCLP1 and HuCCT1 cell (*P* < 0.001, Fig. 3A).

In transfected cells, miR-451 overexpression markedly suppressed cell proliferation, and miR-451 knockdown notably promoted CCLP1 and HuCCT1 cell proliferation (*P* < 0.05, *P* < 0.01, Fig. 3B). Additionally, the migration of CCLP1 and HuCCT1 cells was also inhibited by miR-451 overexpression and accelerated by the silencing of miR-451 (*P* < 0.001, Fig. 3C, Fig. S1). Similarly, the overexpression of miR-451 repressed the invasion of CCLP1 and HuCCT1 cells and the miR-451 knockdown showed a dramatically enhanced effect on cell invasion of cholangiocarcinoma (*P* < 0.001, Fig. 3D, Fig. S1).

### miR-451 negatively regulates the expression of ATF

**Fig. 4:**
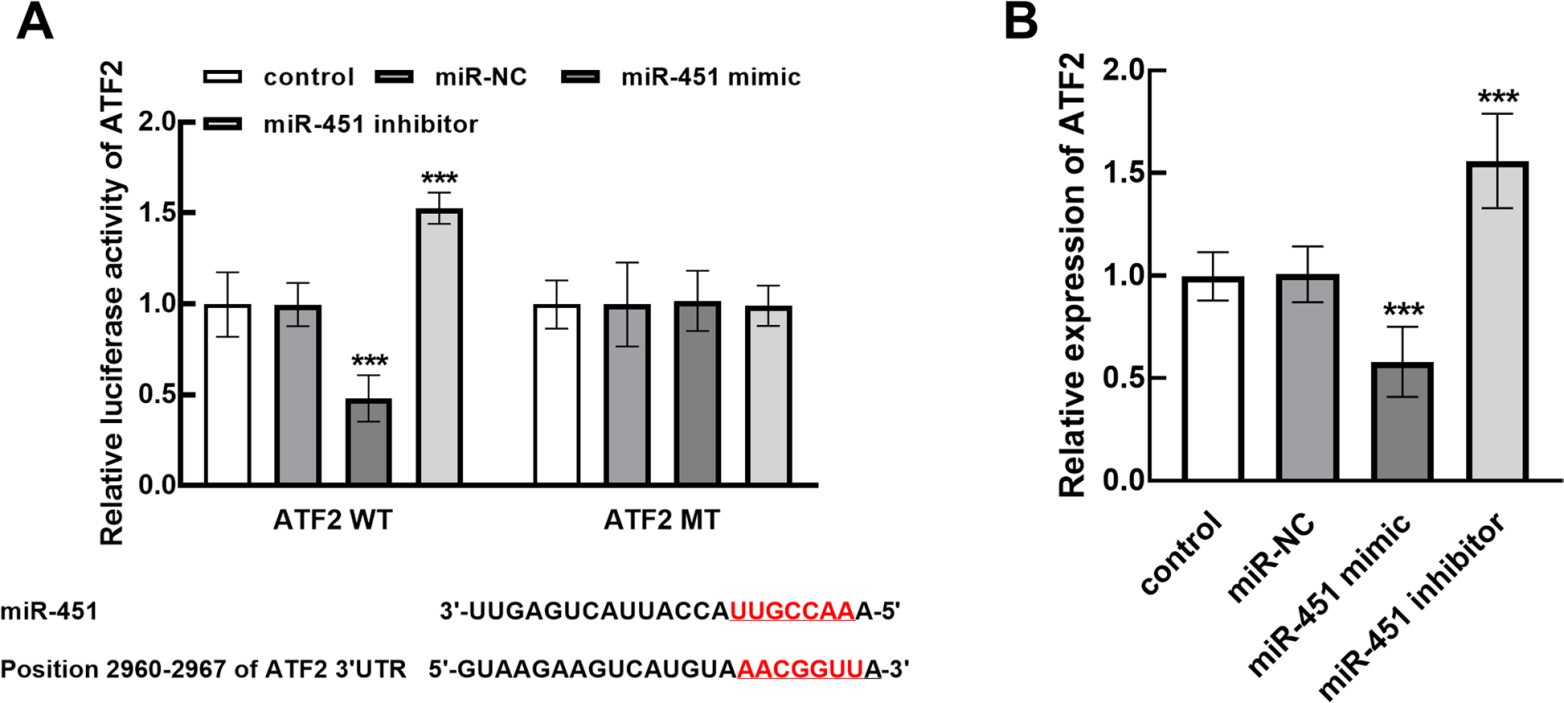
Regulatory effect of miR-451 on ATF2 evaluated by luciferase reporter (**A**) and PCR (**B**) in CCLP1 cell. ATF2 was found to bind with miR-451 with several binding sites and was negatively regulated by miR-451. ***P < 0.001

ATF2 was predicted to bind with miR-451 with several binding sites, and the luciferase of ATF2 was suppressed by the overexpression of miR-451 and enhanced by miR-451 knockdown (Fig. 4A). While the expression of ATF2 was also negatively regulated by miR-451 (Fig. 4B).

## Discussion

Significant dysregulation of miRNAs in tumors always insinuates their potential functional roles in human diseases. miR-451 has been widely reported to possess abnormal expression and participate in the progression of human diseases. For example, miR-451 was identified as the most strongly downregulated miRNA in non-small cell lung cancer (NSCLC) and showed significant association with poor differentiation, advanced clinical stage, and positive lymph node metastasis of patients [14]. The abnormal expression of miR-451 was observed in colorectal cancer, gastric cancer, and bladder carcinoma [15–17]. miR-451 has been revealed to be downregulated in hepatocellular carcinoma (HCC) and was involved in the tumor progression and disease development of patients [18]. Both HCC and cholangiocarcinoma are derived from the substance of the hepatic parenchyma and are known as primary liver cancer [19]. In a previous study, miR-451 was demonstrated as a downregulated miRNA in cholangiocarcinoma [8]. miR-451 was speculated to be involved in the pathogenesis and development of cholangiocarcinoma, which lacked available data.

The consistent downregulation of miR-451 in the cholangiocarcinoma was observed in the present study, and its significant association with TNM stage and lymph node metastasis status of patients, two major indicators of cholangiocarcinoma progression, was also dugout, suggesting its involvement in cholangiocarcinoma development. miR-451 was also demonstrated to participate in the development of many other cancers for its close relationship with the clinicopathological characteristics of patients. For instance, miR-451 was significantly correlated with the FIGO stage and lymph node metastasis of ovarian cancer patients, and it also predicted patients’ poor prognosis, indicating its significance in cancer progression and prognosis [20]. The significant association between miR-451 and lymph node metastasis was also observed in thyroid cancer and miR-451 was remarkably upregulated in lymph node metastasis tissues compared with tissues without lymph node metastasis [21]. The diagnostic value of miR-451 has been illustrated in various human solid tumors in previous studies, such as gastric cancer, breast cancer, and renal cell carcinoma [22–24]. miR-451 was also revealed to predict the recurrence of colorectal cancer and gastric cancer [25, 26]. Here, the downregulation of miR-451 could also differentiate cholangiocarcinoma patients from healthy volunteers, indicating that miR-451 could as serve as a diagnostic biomarker of cholangiocarcinoma. While the function of miR-451 in the prediction of cholangiocarcinoma recurrence needs further representative samples to estimate.

Previously, miR-451 was disclosed to induce G0/G1 phase arrest and the apoptosis of glioblastoma, but the molecular mechanism was controversial [27, 28]. The inhibitory effect of miR-451 on cellular processes of osteosarcoma was revealed [29]. *In vitro*, the dysregulation of miR-451 affected the proliferation, migration, and invasion of cholangiocarcinoma cells. Specifically, the miR-451 overexpression inhibited cell growth, migration, and invasion, whereas the knockdown of miR-451 promoted the cellular processes of cholangiocarcinoma. These results leaked out that miR-451 functioned as a tumor suppressor during the progression of cholangiocarcinoma.

Although the clinical significance and biological function of mir-451 has been revealed, the concrete mechanism underlying these functional roles is also an important part. Several molecules have been demonstrated as the direct targets of miR-451 during its biological function in many other tumors. For example, PGE2 has been reported to mediate the inhibition of osteosarcoma cellular processes by miR-451, and it was found to promote the development of cholangiocarcinoma [30, 31]. ATF2 was found to be negatively regulated by miR-451 through the results of luciferase reporter and expression validation, which is consistent with previous studies [32]. Therefore. ATF2 was speculated to mediate the suppressor role of miR-451 in cholangiocarcinoma.

However, the identification of a single miRNA biomarker neglects the potential of other miRNAs with high scores. Recently, the establishment of miRNA signatures has become a research hot point in cancer research. Therefore, future studies would focus on the significance of miR-451 combining with other miRNAs to establish potential signatures.

## Conclusions

In conclusion, downregulated miR-451 in cholangiocarcinoma showed a close association with the disease development and clinical prognosis. Additionally, miR-451 could distinguish cholangiocarcinoma patients from healthy volunteers with high specificity and sensitivity and it also acted as a tumor suppressor that negatively regulated the proliferation, migration, and invasion of cholangiocarcinoma cells.

## Methods

### Patients and samples

This study was performed in line with the principles of the Declaration of Helsinki. Approval was granted by the Ethics Committee of Mindong Hospital  Affiliated to Fujian Medical University. One hundred and fifty-nine patients diagnosed with cholangiocarcinoma and sixty-four healthy volunteers who received routine physical examinations at Mindong Hospital of Ningde City were included in this study during 2013–2015. The serum samples, tumor tissues, and matched normal tissues were collected after receiving informed consent from every participator. While only the serum samples were collected from healthy individuals. The cholangiocarcinoma patients were followed up for five years to obtain their survival status after surgery.

### Quantitative Real-Time Polymerase Chain Reaction (qRT-PCR)

It is a two-step process of miR-451 expression assessment. Total RNA was isolated and used to synthesize cDNA with the TaqMan Advanced miRNA cDNA Synthesis Kit (Thermo Fisher Scientific, USA). cDNA was diluted and mixed with the SYBR Green master reagent and primer mix. The PCR process was performed with an ABI 7500 system (Applied Biosystems, USA). The 2^−ΔΔCt^ method was used to calculate the relative expression of miR-451 with GAPDH as the internal reference.

### Cell culture and cell transfection

Cholangiocarcinoma cell lines (CCLP1, HuCCT1, SNU1196, and KKU-100 cells, ATCC) and normal cholangiocyte H69 cells (ATCC) were cultured in a DMEM culture medium. Cell culture was conducted in a constant temperature incubator at 37°C with 5% CO_2_. Cells reached the logarithmic period were transfected with miR-451 mimic (5’-AAACCGUUACCAUUACUGAGUU-3’), miR-451 inhibitor (5’-AACUCAGUAAUGGUAACGGUUU-3’), or corresponding negative controls (mimic NC and inhibitor NC) with the help of Lipofectamine 2000 (Invitrogen, USA).

### Cell proliferation assay

Cells (1× 10^5^ cells/well) were seeded into 96-well plates and incubated with DMEM culture medium for a specific period. Then, the CCK8 reagent was added to each well and incubated with the mixture for 1 h. OD450 of each well was detected with the employment of a microplate reader (Thermo Fisher Scientific, USA). The experiments were performed three times to obtain the mean values.

### Cell migration and invasion assay

A total of 2× 10^4^ cells/well were seeded into the upper chamber of the 24-well transwell chambers with a pore size of 8 µm (Corning, USA). The upper chamber was supplied with a serum-free culture medium, while the FBS-containing medium was placed in the bottom chamber. The chambers were incubated at 37°C for 24 h, and the migrated and invaded cells on the lower surface were fixed and stained. The number of cells was counted with the help of a microscope (Olympus, Japan).

### Luciferase reporter assay

The wild-type vector was established by cloning the binding sites between miR-451 and ATF2, while the mutant-type vector was constructed with the point mutations. The vectors were co-transfected with miR-451 mimic, inhibitor, or negative controls into the CCLP1 cell, and the relative luciferase activity of ATF2 was detected after 48 h of transfection using the Dual-luciferase repoter Assay System (Promega, USA).

### Statistical analysis

All data were represented as mean value ± standard deviation obtained from at least three independent experiments. The difference between groups was analyzed by the student’s t-test and one-way ANOVA.

The difference in the expression of miR-451 between healthy volunteers and cholangiocarcinoma was used to evaluate the diagnostic value of miR-451 with the help of the ROC curve. While the prognostic value of miR-451 was assessed with the Kaplan-Meier and Cox regression analysis. *P* < 0.05 was considered to be statistically significant.

## Supplementary Information

**Additional file 1:** **Figure S1.** Representative images of Transwell assay inevaluating cell migration and invasion.

## Data Availability

The datasets used and/or analysed during the current study are available from the corresponding author on reasonable request.

## Declarations

The serum samples, tumor tissues, and matched normal tissues were collected after receiving informed consent from every participator.
