# Supplementary figures and images for: Downregulation of miR-451 in cholangiocarcinoma help the diagnsosi and promotes tumor progression
Source: BMC Mol Cell Biol. 2022 Nov 9;23:46. doi: 10.1186/s12860-022-00445-2 (PMC9647969; doi:10.1186/s12860-022-00445-2)

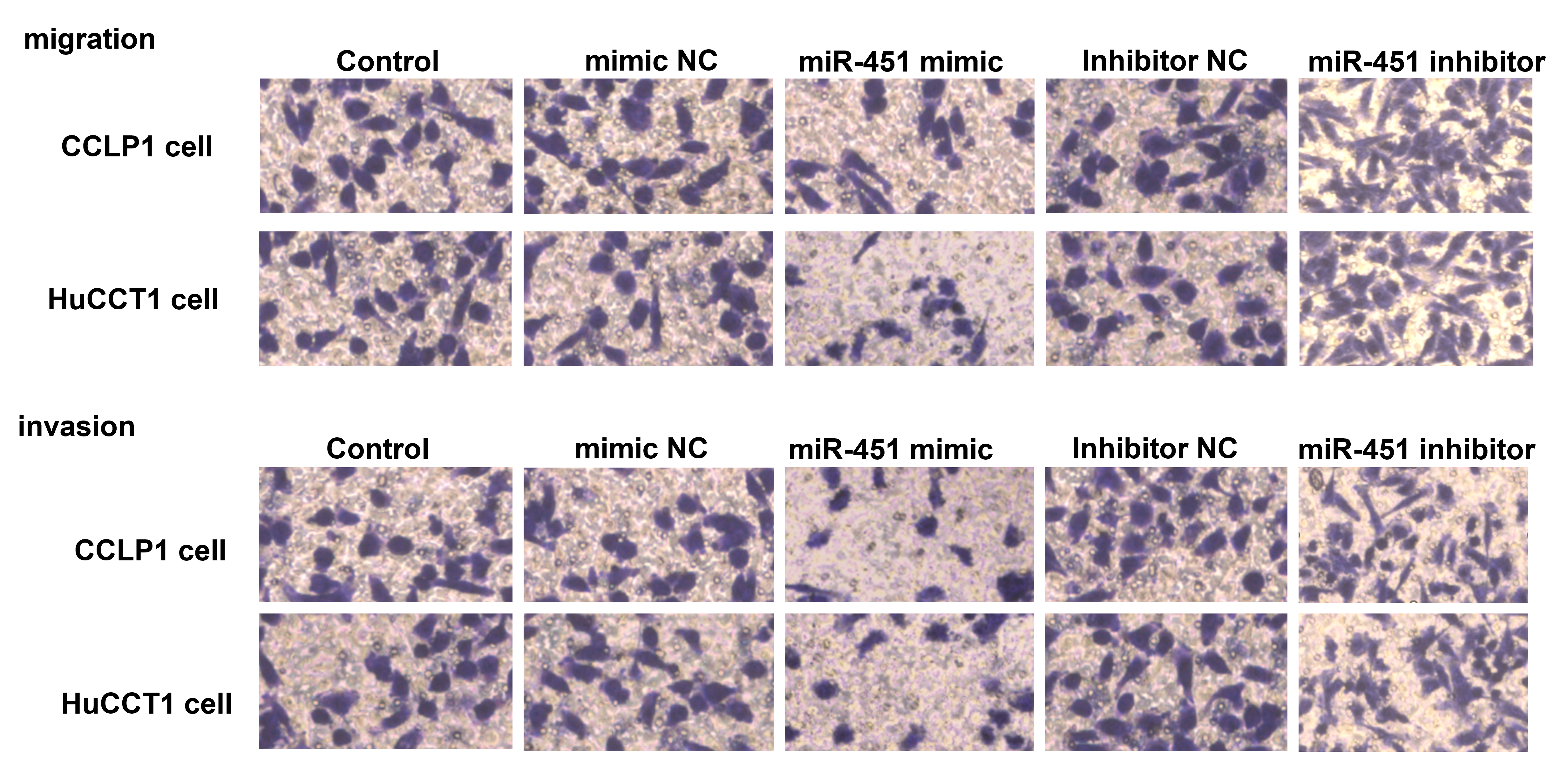

Supplement: Supplementary file 1 — Additional file 1: Figure S1. Representative images of Transwell assay inevaluating cell migration and invasion. [file 12860_2022_445_MOESM1_ESM.tif]
